# Mediating role of malnutrition in the associations between oral frailty, food insecurity, and depression among rural older adults: an exploratory model-based analysis

**DOI:** 10.3389/fpubh.2026.1852515

**Published:** 2026-07-31

**Authors:** Nurhayat Tugra Ulema

**Affiliations:** Department of Nutrition and Dietetics, Faculty of Health Sciences, Ağrı İbrahim Çeçen University, Agri, Türkiye

**Keywords:** biopsychosocial model, depression, food insecurity, malnutrition, oral frailty, rural health

## Abstract

**Background:**

Depression is a growing concern among rural older adults and is associated with factors such as oral frailty and food insecurity, with malnutrition possibly contributing to these associations. This study aimed to examine malnutrition as a potential statistical mediator in the associations between oral frailty, food insecurity, and depression within a biopsychosocial perspective, using an exploratory, model-based approach.

**Methods:**

This cross-sectional study enrolled 327 community-dwelling older adults from three rural provinces of Türkiye. Oral frailty was assessed using the Oral Frailty Index-8 (OFI-8), food insecurity using the Food Insecurity Experience Scale (FIES), malnutrition using the Mini Nutritional Assessment-Short Form (MNA-SF), and depression using the Geriatric Depression Scale-30 (GDS-30). Multiple logistic regression and bootstrap mediation analyses (5,000 resamples), with malnutrition as the hypothesized mediator, were performed adjusting for age, gender, income, living arrangement, and IADL score.

**Results:**

The median age was 64 [interquartile range (IQR): 61–70] years, 58.4% were female. Depressive symptoms were identified in 34.9% of participants. Oral frailty [OR (95% CI) = 1.964 (1.452–2.658), *p* < 0.001], food insecurity [OR (95% CI) = 1.381 (1.066–1.790), *p* = 0.015], and malnutrition [OR (95% CI) = 1.949 (1.538–2.469), *p* < 0.001] were significantly associated with depression after adjusting for potential confounders (age, gender, living arrangement, income, IADL score). Mediation analysis revealed that malnutrition mediated approximately 23.6% of the total effect of oral frailty on depression (total effect *β* = 0.055, 95% CI: 0.025–0.087, *p* = 0.002) and 22.0% of the total effect of food insecurity (total effect *β* = 0.050, 95% CI: 0.020–0.085, *p* = 0.002).

**Conclusion:**

Malnutrition significantly associated with oral frailty, food insecurity, and depression in rural older adults. Integrated interventions targeting nutrition, oral health, and food security may be beneficial in mitigating depression risk in this vulnerable population.

## Introduction

Depression is a significant public health issue among older adults, particularly in rural populations, where it profoundly impacts quality of life, physical health, and psychological well-being. Recent epidemiological data indicate prevalence rates between 23.6 and 45.8% in rural older adult populations, a variation often attributed to geographic disparities and socioeconomic constraints (1–4). This elevated burden is increasingly understood through the lens of ‘systemic vulnerability’ — a multidimensional state where cumulative declines across biological, functional, and social domains reduce an individual’s resilience to environmental stressors (5). Within this framework, rural older populations face a unique synergy of challenges where physical impairments and resource limitations do not exist in isolation but rather coalesce to exacerbate mental health risks.

The biopsychosocial model offers a comprehensive framework for understanding depression through the interplay of biological and social factors (6). Within this context, oral frailty and food insecurity emerge as critical yet distinct factors that warrant further investigation regarding their contribution to geriatric depression. Oral frailty is recognized as a multidimensional construct involving a cumulative decline in oral health and function; while characterized by diminished functions such as tooth loss, chewing difficulty, and dysphagia, it also encompasses broader physiological and psychosocial impairments (7). The mechanism through which oral frailty transitions into systemic frailty often involves compromised mastication, which directly impairs nutrient intake and triggers a cascade of functional deterioration (8–10). Similarly, food insecurity, defined as limited or uncertain availability of nutritionally adequate and safe foods, is a documented risk factor for psychological distress (11). In rural contexts, this is compounded by ‘food deserts’ and limited transportation, creating a dual burden: a biological deficit from poor nutrient density and a psychological burden marked by chronic anxiety over food availability (12, 13).

Malnutrition may represent an important biological factor through which structural and functional vulnerabilities become linked to mental health outcomes. Biologically, nutritional deficiencies—particularly in B vitamins, omega-3 fatty acids, and essential amino acids—disrupt the synthesis of key neurotransmitters such as serotonin and dopamine, ultimately altering neural plasticity and increasing susceptibility to geriatric depression (14). Cognitively, inadequate nutrient density appears to be associated with declines in executive function and processing speed; in older adults, this may manifest as diminished mental resilience, impairing the ability to employ effective coping mechanisms against environmental stressors and thereby contributing to depressive symptoms (15). Socially, malnutrition may result in physical wasting and frailty, which can lead to social withdrawal. In rural settings, where communal eating is a cornerstone of social engagement, the inability to participate due to poor health may further intensify social isolation—a well-documented correlate of late-life depression (16, 17). However, despite growing recognition of these relationships, the potential role of malnutrition in the associations between oral frailty, food insecurity, and depression remains poorly understood in rural older populations. This gap exists primarily due to a lack of integrated, model-based approaches that simultaneously examine these associations, combined with limited rural-focused data exploring these co-occurring relationships in resource-limited settings.

To address these gaps, this study explored whether malnutrition may serve as a potential intermediary in the associations between oral frailty and depression, and between food insecurity and depression, among community-dwelling rural older adults in Türkiye, within a biopsychosocial framework. We hypothesized that malnutrition might partially account for both associations, representing a shared biological factor through which structural and functional vulnerabilities may co-occur with depressive symptoms. Using bootstrap mediation analysis within an exploratory, model-based framework, this study aims to inform integrated geriatric intervention strategies that simultaneously target nutritional status, oral health, and food security in rural settings.

## Methods

### Study setting and participants

This cross-sectional study was conducted in three rural provinces of Turkiye: Agrı, Kars and Mardin. These three specific provinces (Ağrı, Kars, and Mardin) were intentionally selected due to their unique geographical and socio-economic characteristics in the eastern and southeastern regions of Türkiye. Ağrı and Kars are characterized by high altitudinal plateaus and extremely harsh, prolonged winter conditions, which frequently restrict logistics and sustainable access to fresh, diverse food options for rural older populations. Mardin, while geographically distinct, shares highly parallel socio-economic disadvantages, high household crowding indexes, and a rapid concentration of the older adult population due to younger demographics migrating to western urban centers. Selecting these provinces allows for a comprehensive understanding of biological and structural vulnerabilities in resource-limited rural environments of regional Anatolia. A convenience sampling method was employed, with recruitment conducted exclusively in community-based settings frequently visited by older adults. These included rural community centers, local neighborhood gathering places, and social centers. Eligible participants were community-dwelling individuals aged 60 years and older. Individuals with a diagnosis of active malignancy, Alzheimer’s disease, or those receiving enteral/parenteral nutritional support were excluded. The minimum sample size was calculated as 128 using *G*Power 3.1.9.7 software, based on logistic regression with an expected odds ratio of 2.5, an anticipated depression prevalence of 30% based on previous literature (18, 19), a power (1-*β*) of 0.95, and an alpha level of 0.05, with depressive symptoms (GDS-30) as the primary outcome. The final sample of 327 participants exceeded this minimum threshold, providing sufficient statistical power for both the regression and bootstrap-based mediation analyses conducted.

### Data collection and measures

#### Sociodemographic and clinical characteristics

Data on age, gender, marital status, education level, income, smoking and alcohol use, chronic diseases, and current medication use were collected via self-report. The sociodemographic and clinical characteristics were collected using a researcher-developed data collection form intended solely for descriptive purposes and not as a standardized questionnaire. Participants were approached face-to-face by trained researchers during daytime hours at rural community centers, local neighborhood gathering places (kahvehanes), and public social hubs. Recruitment was carried out on a voluntary basis. Out of 355 eligible older adults initially approached, 28 declined to participate or complete the interview, resulting in a recorded refusal rate of 7.8%. Given the non-probability convenience sampling framework, recruitment did not follow a consecutive multi-stage clinical registry. While this pragmatic approach was necessitated by the logistical and geographical constraints of remote rural settings, the potential implications of this sampling method on generalizability and selection parameters are explicitly addressed within the study’s limitations.

#### Anthropometric measurements

Body weight was measured using a calibrated digital scale (accuracy: 0.1 kg) with participants fasting and wearing light clothing. Height was measured using a wall-mounted stadiometer (accuracy: 0.1 cm), with participants standing barefoot in the Frankfurt horizontal plane. Body mass index (BMI) was calculated as weight (kg) divided by height squared (m^2^).

#### Functional dependency

Functional status was assessed using the Activities of Daily Living (ADL) and Instrumental Activities of Daily Living (IADL) indices. Dependency was considered as ADL score<6 (20), IADL score<8 (21).

#### Oral frailty assessment

Oral frailty was measured using the Oral Frailty Index-8 (OFI-8), an eight-item questionnaire assessing oral health status and function (e.g., tooth loss, chewing difficulties, swallowing problems). The total score of the OFI-8 ranges between 0 and 11, with scores above 4 suggesting the presence of oral frailty (22). The Turkish version of the instrument has been previously validated for use in older adults, demonstrating stable clinical utility and strong convergence with nutritional and physical frailty parameters (23). In the present study, the OFI-8 yielded good internal consistency, with a Cronbach’s alpha coefficient of 0.77.

#### Food insecurity assessment

Food insecurity was assessed using the Food Insecurity Experience Scale (FIES), a standardized tool developed by the Food and Agriculture Organization (FAO) to measure access to adequate food of individuals. The scale consists of eight questions reflecting increasing severity of food insecurity, with total raw scores ranging from 0 to 8 (24). Since the FIES does not have universally fixed cut-off points (25), raw score-based discrete classification was applied solely for descriptive purposes, following thresholds proposed in previous studies (26): 0 = food secure; 1–3 = mild food insecurity; 4–8 = moderate-to-severe food insecurity. The latter two categories were combined due to the limited number of participants with severe food insecurity. The psychometric properties and cultural appropriateness of the FIES within the Turkish context have been established in previous research (27, 28). In the current sample, the scale demonstrated strong internal consistency, with a Cronbach’s alpha coefficient of 0.75.

#### Nutritional assessment

The nutritional status of the study participants was evaluated using the Mini Nutritional Assessment–Short Form (MNA-SF). Responses were obtained from both the participants and their caregivers. In cases where participants were uncertain about specific items, the information was provided by the caregiver. Scores were interpreted as: 0–7 = malnutrition; 8–11 = risk of malnutrition; 12–14 = normal nutritional status (29). The validated Turkish adaptation of the MNA-SF was used throughout the study (30). In this geriatric sample, the instrument demonstrated satisfactory internal consistency, with a Cronbach’s alpha coefficient of 0.84.

#### Depression assessment

Depression status was assessed using the Geriatric Depression Scale (GDS-30), a widely used self-report screening tool designed to identify depressive symptoms in older adults. The scale consists of 30 yes/no items that evaluate mood, cognitive, and somatic symptoms related to depression. Total scores range from 0 to 30, with higher scores indicating greater depressive symptomatology. Scores between 0 and 9 are considered normal, 10 to 19 indicate mild depression, and 20 to 30 indicate severe depression (31). For the purposes of this study, participants with GDS-30 scores ≥10 were classified as having depression. The Turkish validation of the GDS-30 established by Ertan and Eker (32) was employed, and the scale demonstrated robust internal consistency in this sample, with a Cronbach’s alpha coefficient of 0.86.

### Statistical analysis

All statistical analyses were conducted using R software version 4.3.1 (R Foundation for Statistical Computing, Vienna, Austria). Continuous variables are presented as medians with interquartile ranges (IQR), while categorical variables are expressed as frequencies and percentages. Group comparisons based on depression status were performed using the Mann–Whitney U test for continuous parameters and Chi-square tests for categorical variables. Independent predictors of late-life depression were identified through multiple logistic regression models adjusting for potential confounders (age, gender, income, living arrangement, and IADL score) selected *a priori* based on established geriatric depression literature. The results of the regression models are reported as odds ratios (OR) and 95% confidence intervals (CI).

To explore potential underlying associations, two separate simple mediation analyses were conducted using the non-parametric bootstrapping approach (with 5,000 resamples) via the ‘mediation’ package in R, to explore whether malnutrition may serve as a potential statistical intermediary in the associations between (a) oral frailty and depression, and (b) food insecurity and depression, within an exploratory, model-based framework. All mediation models were adjusted for the same baseline sociodemographic confounders. Prior to conducting the mediation models, key structural assumptions were evaluated; linearity was verified via residual plots, and multicollinearity diagnostics confirmed that the Variance Inflation Factors (VIF) for the main exposures ranged between 1.34 and 2.05, well below the conservative threshold of 5.0, indicating no significant multicollinearity concern.

For the mediation framework, depression was operationalized as a continuous outcome using the total GDS-30 score, rather than the binary classification used in the primary logistic regression models. This linear approach aligns with methodological recommendations for regression-based mediation frameworks (33), preserves the continuous spectrum of depressive symptom severity, and avoids the statistical information and power loss inherent in artificial dichotomization, thereby enhancing the precision of the estimated indirect coefficients. The indirect and direct statistical estimates are reported as unstandardized β coefficients. Finally, to formally address the inherent limitations of establishing mediation with cross-sectional data, non-parametric sensitivity analyses evaluating potential violations of the sequential ignorability assumption were performed using the medsens function within the R ‘mediation’ package, supporting the consistency of the reported indirect associations within the cross-sectional design. A two-tailed *p*-value of less than 0.05 was considered statistically significant.

## Results

A total of 327 community-dwelling older adults were included in the study. The median age of participants was 64 years [interquartile range (IQR): 61–70], and 58.4% were female. The median GDS-30 score was 8 (IQR: 5–16). Based on GDS-30 thresholds, 114 participants (34.9%) were classified as having depression, 88 with mild and 26 with severe depression. These severity categories were used solely for descriptive characterization of the sample and were not incorporated into the regression or mediation analyses. The remaining 213 participants (65.1%) were classified as having no depressive symptoms. The median GDS-30 score was 14 (IQR: 12–17) among those with depressive symptoms and 5 (IQR: 3–7) among those without.

Participants with depressive symptoms were significantly older than those without, with median ages of 65 and 61 years, respectively (*p* < 0.001). Participants with depressive symptoms included a significantly higher proportion of females (*p* < 0.001). Additionally, participants with depressive symptoms had significantly lower education levels, were more likely to be divorced or widowed, more frequently lived alone, and reported lower income levels compared to those without depressive symptoms (*p* < 0.001 for all comparisons; Table 1).

**Table 1:** Sociodemographic characteristics of the participants.

Variable	Overall (*n* = 327)	Depression (+) (*n* = 114)	Depression (−) (*n* = 213)	*p*
Age, years	64 (61–70)	65 (61–73)	61 (60–65)	**<0.001**
Gender, *n* (%)				
Male	136 (41.6)	35 (30.7)	101 (47.4)	**<0.001**
Female	191 (58.4)	79 (69.3)	112 (52.6)	
BMI, kg/m^2^	28.1 (24.9–32.0)	28.6 (25.4–32.1)	28.1 (24.7–32.0)	0.501
Education, *n* (%)				
Not literate	142 (43.4)	64 (56.1)	78 (36.7)	**<0.001**
Primary school	89 (27.2)	23 (20.2)	66 (31.0)	
Middle school	40 (12.2)	11 (9.6)	29 (13.6)	
High school	56 (17.1)	16 (14.0)	40 (18.8)	
Marriage status, *n* (%)				
Married	255 (78.0)	83 (72.8)	172 (80.8)	**<0.001**
Single	4 (1.2)	1 (0.9)	3 (1.4)	
Divorced/Widowed	68 (20.8)	30 (26.3)	38 (17.8)	
Employment status, *n* (%)				
Employed	59 (18.0)	35 (30.7)	24 (11.3)	**<0.001**
Unemployed	198 (60.6)	50 (43.9)	148 (69.5)	
Retired	70 (21.4)	29 (25.4)	41 (19.2)	
Living arrangement				
Living alone	50 (15.3)	31 (27.2)	19 (8.9)	**0.003**
Living with partner	71 (21.7)	12 (10.5)	59 (27.7)	
Living with partner and children	206 (63.0)	71 (62.3)	135 (63.4)	
Income status, *n* (%)				
Income > Expenses	54 (16.5)	18 (15.8)	36 (16.9)	**0.001**
Income = Expenses	168 (51.4)	51 (44.7)	117 (54.9)	
Income < Expenses	105 (32.1)	45 (39.5)	60 (28.2)	
Smoking status, *n* (%)				
Yes	72 (22.0)	30 (26.3)	42 (19.7)	0.170
No	255 (78.0)	84 (73.7)	171 (80.3)	
Alcohol status, *n* (%)				
Yes	13 (4.0)	5 (4.4)	8 (3.8)	0.788
No	314 (96.0)	109 (95.6)	205 (96.2)	

Comparisons between the Depression (+) and Depression (−) groups were performed using the Mann–Whitney U test for continuous variables and the chi-square test for categorical variables. Statistical significance was defined as *p* < 0.05.

Functional dependency was significantly more prevalent among participants with depression, as indicated by both ADL (*p* < 0.001) and IADL scores (*p* = 0.010). Participants with depression had significantly lower MNA-SF scores compared to those without depression [11 (IQR: 9–12) vs. 14 (IQR: 12–14); *p* < 0.001]. Based on nutritional status categories, the prevalence of malnutrition and risk of malnutrition was significantly higher among the depressed group compared to the non-depressed group (*p* < 0.001).

The Oral Frailty Index-8 (OFI-8) scores were higher among the depressed group [median: 4 (IQR: 3–5) vs. 2 (IQR: 2–3); *p* < 0.001]. Food insecurity, as measured by the Food Insecurity Experience Scale (FIES), was also higher among those with depression [median: 0 (IQR: 0–3) vs. 0 (IQR: 0–1); *p* < 0.001], with mild and moderate to severe food insecurity rates of 10.5 and 4.4%, respectively, compared to 6.1 and 1.4% in the non-depressed group (*p* < 0.001; Table 2).

**Table 2:** Clinical characteristics of the study population.

Variable	Overall (*n* = 327)	Depression (+) (*n* = 114)	Depression (−) (*n* = 213)	*p*
Comorbidities, *n* (%)				
Hypertension	120 (36.7)	49 (43.0)	71 (33.3)	**0.002**
Diabetes mellitus	65 (19.9)	26 (22.8)	39 (18.3)	0.053
Cardiovascular disease	38 (11.6)	15 (13.2)	23 (10.8)	0.240
Pulmonary disease	9 (2.8)	5 (4.4)	4 (1.9)	0.420
Chronic kidney disease	13 (4.0)	11 (9.6)	2 (0.9)	**0.036**
Malignancy	8 (2.4)	5 (4.4)	3 (1.4)	0.549
Dyslipidemia	9 (2.8)	5 (4.4)	4 (1.9)	0.541
Hypothyroidism	4 (1.2)	1 (0.9)	3 (1.4)	0.090
Dementia	4 (1.2)	4 (3.5)	–	0.141
Parkinson’s disease	1 (0.3)	1 (0.9)	–	0.464
ADL score	6 (6–6)	6 (5–6)	6 (6–6)	**<0.001**
ADL Dependency, *n* (%)	65 (19.9)	31 (27.2)	34 (15.6)	**<0.001**
IADL score	6 (4–8)	6 (4–7)	6 (5–8)	**0.010**
IADL Dependency, *n* (%)	245 (74.9)	92 (80.7)	153 (71.8)	**0.002**
MNA-SF score	12 (10–14)	11 (9–12)	14 (12–14)	**<0.001**
Nutritional status, *n* (%)				
Malnutrition	23 (7.0)	12 (10.5)	11 (5.2)	**<0.001**
At risk of malnutrition	132 (40.4)	68 (59.6)	64 (30.0)	
Well-nourished	172 (52.6)	34 (29.8)	138 (64.8)	
OFI-8 index score	3 (2–4)	4 (3–5)	2 (2–3)	**<0.001**
Oral frailty status				
Oral frail	186 (56.9)	70 (61.4)	116 (54.5)	0.224
Non-oral frail	141 (43.1)	44 (38.6)	97 (45.5)	
FIES score	0 (0–2)	0 (0–3)	0 (0–1)	**<0.001**
Food security status				
Food secure	294 (89.9)	97 (85.1)	197 (92.5)	**<0.001**
Mild FI	25 (7.6)	12 (10.5)	13 (6.1)	
Moderate to Severe FI	8 (2.4)	5 (4.4)	3 (1.4)	

ADL, Activities of Daily Living; IADL, Instrumental Activities of Daily Living; FI, Food Insecure; FIES, Food Insecurity Experience Scale; MNA-SF, Mini Nutritional Assessment-Short Form; OFI, Oral Frailty Index. Comparisons between the Depression (+) and Depression (−) groups were performed using the Mann–Whitney U test for continuous variables and the chi-square test for categorical variables. Statistical significance was defined as *p* < 0.05; bold values indicate statistically significant results.

After adjusting for potential confounders (age, gender, living arrangement, income, and IADL score), all three exposures remained independently associated with depression.

According to the multiple logistic regression models, the continuous MNA-SF score emerged as a significant independent predictor of late-life depression. Specifically, each one-point decrease in the MNA-SF score (reflecting a worsening nutritional status) was associated with a 1.949-fold increase in the likelihood of depression (OR = 1.949, 95% CI: 1.538–2.469, *p* < 0.001). Higher OFI-8 scores were similarly associated with increased odds of depression [OR = 1.964, 95% CI: 1.452–2.658; p < 0.001]. Elevated food insecurity levels were also significantly associated with a greater likelihood of depression [OR = 1.381, 95% CI: 1.066–1.790; *p* = 0.015] (Table 3).

**Table 3:** Logistic regression analysis of malnutrition, oral frailty, and food insecurity in predicting depression.

Variable	Crude		Adjusted	
	OR (95% CI)	*p*-value	OR (95% CI)	*p*-value
MNA-SF score	2.119 (1.692–2.660)	**<0.001**	1.949 (1.538–2.469)	**<0.001**
OFI-8 score	2.065 (1.553–2.747)	**<0.001**	1.964 (1.452–2.658)	**<0.001**
FIES score	1.343 (1.059–1.703)	**0.015**	1.381 (1.066–1.790)	**0.015**

*p*-values were calculated using multiple logistic regression analysis, adjusted for age, gender, living arrangement, income, and IADL score. Statistical significance was defined as *p* < 0.05; bold values indicate statistically significant results. For the MNA-SF score, the odds ratios represent the change in odds of depression associated with a one-unit decrease in the MNA-SF score (i.e., worsening nutritional status). For the OFI-8 and FIES scores, the odds ratios correspond to a one-unit increase in the respective scores (i.e., greater oral frailty and higher food insecurity).

Exploratory mediation analysis indicated that oral frailty had a statistically significant total association with depression (*β* = 0.055, 95% CI: 0.025–0.087, *p* = 0.002). Malnutrition statistically functioned as a partial intermediary in this association (estimated indirect association: *β* = 0.013, 95% CI: 0.004–0.023, *p* = 0.009), accounting for an estimated 23.6% of the total association. The direct association between oral frailty and depression remained statistically significant after accounting for malnutrition (*β* = 0.042, 95% CI: 0.015–0.073, *p* = 0.005).

**Figure 1:**
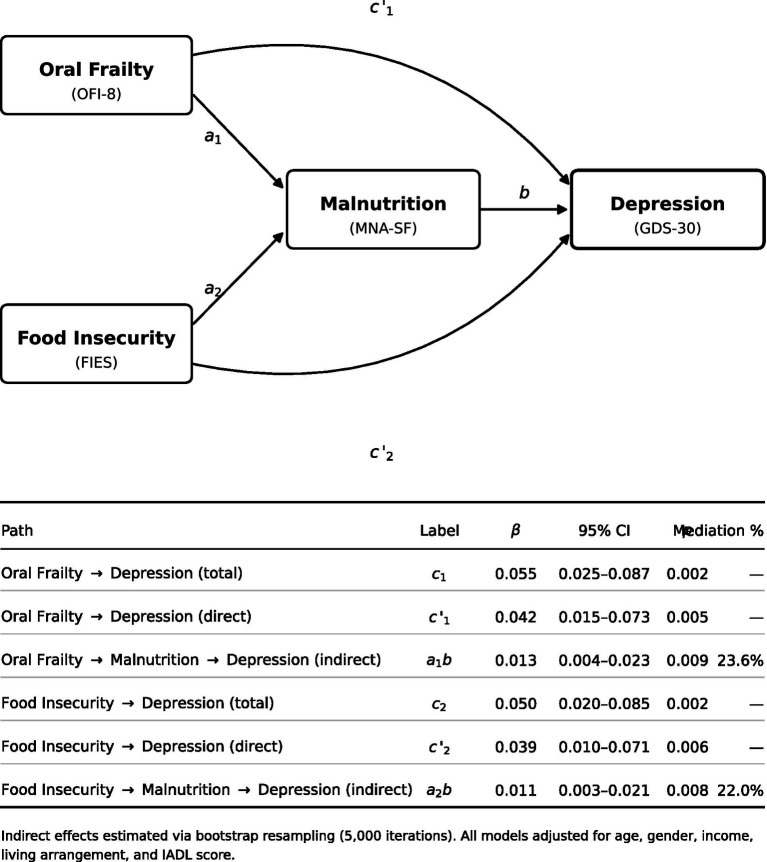
Exploratory model-based indirect associations of malnutrition in the relationships between food insecurity, oral frailty, and depression. Figure created by the author with the assistance of Claude (Anthropic); reviewed and verified by the author.

Similarly, food insecurity showed a significant total association with depression (*β* = 0.050, 95% CI: 0.020–0.085, *p* = 0.002), with malnutrition accounting for an estimated 22.0% of this concurrent association (estimated indirect association: *β* = 0.011, 95% CI: 0.003–0.021, *p* = 0.008). The direct association also remained statistically significant (*β* = 0.039, 95% CI: 0.010–0.071, *p* = 0.006). These exploratory, model-based findings are illustrated in Figure 1.

## Discussion

This cross-sectional study explored the potential role of malnutrition in the associations between oral frailty, food insecurity, and depression among community-dwelling older adults residing in three rural provinces of Türkiye, within a biopsychosocial framework. Depressive symptoms were identified in 34.9% of participants. This rate falls within the range reported in community-dwelling older adult samples from rural Türkiye (23.6–45.8%) (4) and is consistent with broader prevalence estimates from rural populations in low- and middle-income countries (1, 2, 4). Logistic regression analyses demonstrated that oral frailty, food insecurity, and malnutrition were each independently associated with depression after adjusting for sociodemographic confounders. Exploratory mediation analyses further indicated that malnutrition accounted for an estimated 23.6% of the total association between oral frailty and depression, and 22.0% of the total association between food insecurity and depression. To our knowledge, this is the first study to simultaneously examine malnutrition as a potential shared intermediary in the associations between oral frailty, food insecurity, and depression within an integrated biopsychosocial framework in a rural older adult population.

Depressive symptoms were more prevalent among women, individuals with lower education levels, those who were divorced or widowed, living alone, or with low income. These demographic patterns align with previous studies highlighting how gender, social disadvantage, and economic vulnerability are linked to poorer mental health outcomes in later life (34, 35). Structural inequalities such as limited access to education, social support, and financial resources may exacerbate biological and behavioral health vulnerabilities, thereby tracking susceptibility to depression. These findings underscore the importance of viewing depression not merely as a consequence of isolated psychosocial stressors but as an outcome of intersecting biological, psychological, and social vulnerabilities — a perspective central to the biopsychosocial framework applied in this study.

Oral frailty, in particular, was significantly associated with depressive symptoms in present study. This finding is directionally consistent with a previous study (36), which reported a 4.89-fold increase in the odds of depression among community-dwelling older adults, although the larger coefficient in that study may reflect differences in population characteristics and assessment methods. Additionally, other studies have identified significant associations between poor oral health and depression in rural older populations (37, 38). Collectively, these findings underscore that oral health does not exist in isolation but concurrently tracks with psychological outcomes among older adults, reinforcing its relevance within integrated geriatric assessments.

Food insecurity was also significantly associated with depression in the present study. A systematic review and meta-analysis reported that food insecurity was associated with a 1.75-fold increased risk of depression across age groups (13), a relationship that may be particularly pronounced in rural older adult populations where food access is further constrained by limited transportation and scarce local resources. In resource-limited settings such as rural Uganda and India, food insecurity rates have been reported to exceed 70%, creating compounding barriers to both physical and mental health (11, 39). While previous international literature from distant settings underscores the severity of food insecurity, our findings highlight how these dynamics unfold within the specific socio-cultural and logistical architecture of eastern Türkiye. Consistent with these observations, malnutrition accounted for an estimated 22.0% of the association between food insecurity and depression in the present study, suggesting that the burden of food insecurity may be partly shared through its concurrent relationship with nutritional status.

The biopsychosocial model offers a more comprehensive framework for interpreting these findings, as it considers how biological risks (e.g., malnutrition), psychological responses (e.g., depression), and social determinants (e.g., food insecurity, oral frailty) interact dynamically. In this study, malnutrition — a key biological factor — was not only independently associated with depression but also both oral frailty and food insecurity and depressive symptoms. This pattern of associations aligns with perspectives in geroscience suggesting that nutritional status may represent an important biological correlate connecting external stressors, functional impairments, and systemic aging processes (40). Recent evidence further suggests that oral health decline, including periodontal disease, is associated with reduced intrinsic capacity in older adults — encompassing nutritional, locomotor, and psychological domains (41) — consistent with the view that oral frailty and malnutrition may represent co-occurring components of a broader biological aging process that tracks with increased vulnerability to depression. Furthermore, these observations are consistent with recent international data; Konda et al. (42) demonstrated a significant positive association between malnourishment and increased depression scores among community-dwelling older adult individuals in India, further highlighting that nutritional depletion represents a global challenge co-occurring with geriatric mental health burden across diverse low- and middle-income rural settings.

From a mechanistic standpoint, oral frailty may relate to nutritional deficiencies through compromised mastication and reduced dietary diversity (8, 9), thereby disrupting the availability of essential amino acids required for neurotransmitter synthesis, including serotonin and dopamine (14). Food insecurity, in turn, may amplify these biological deficits through chronic stress and inadequate nutrient intake (13, 16). That malnutrition accounted for a meaningful proportion of both associations in our exploratory models is consistent with the view that mental health outcomes in rural older adults may be shaped by intersecting vulnerabilities across biological, social, and functional domains — precisely the multilevel perspective the biopsychosocial model was designed to capture.

These findings suggest several potential clinical and policy implications. At the individual level, routine integrated screening for malnutrition, oral frailty, and depressive symptoms could be considered in rural primary care settings, given that each factor independently predicted depression and malnutrition emerged as a potential shared intermediary in our exploratory models. At the system level, expanding food assistance initiatives, integrating geriatric oral health services into rural clinics, and deploying multidisciplinary teams — including dietitians, dentists, social workers, and mental health professionals — may help address the interconnected determinants identified in this study. Community-based social support mechanisms, including peer support groups, caregiver training, and telehealth outreach, may further help mitigate the social isolation that co-occurs with depression risk in remote older populations. Collectively, these multilevel strategies reflect the core principles of the biopsychosocial approach, addressing health holistically through interconnected biological, psychological, and social frameworks.

Several limitations should be acknowledged. First, the use of non-probability convenience sampling may limit the generalizability of the findings to the wider rural older adult population across Türkiye; since the data were collected from three specific rural provinces, these findings may not fully reflect the unique socio-economic and cultural architectures of other geographic regions in the country. Additionally, conducting data collection at public social hubs may have inherently excluded the most functionally impaired, cognitively declined, or homebound older adults who were physically unable to attend community centers, potentially leading to an underestimation of the true prevalence of oral frailty, malnutrition, and depression. Second, although a rigorous statistical mediation framework was employed, the principal methodological constraint of this study is its cross-sectional design, which strictly precludes definitive causal inferences and cannot establish temporal precedence among the investigated variables; consequently, the mediation analyses must be interpreted strictly as exploratory, model-based findings representing associations at a single point in time, rather than confirmed chronological or causal pathways. Furthermore, the assumption of no unmeasured confounding remains an untestable limitation. Despite controlling for key sociodemographic and functional factors in the multi-variable models, residual confounding from unmeasured variables—such as genetic predispositions, specific lifetime dietary patterns, or subclinical chronic inflammation—cannot be completely ruled out, and the findings should therefore be interpreted as robust statistical associations rather than confirmed causal directions. Additionally, the low prevalence of severe food insecurity within the study population limited the capacity for more granular subgroup analyses, potentially reducing statistical power for advanced stratifications. Lastly, reliance on self-reported measures for sensitive constructs, including food insecurity and depressive symptoms, introduces the risk of recall difficulties or social desirability bias among participants, which may affect the subjective estimation parameters.

In conclusion, this study advances the literature by situating malnutrition not as a proven causal factor, but as a potential partial intermediary within an exploratory, model-based cross-sectional framework, in the associations between oral frailty, food insecurity, and depressive symptoms in rural older adults. Grounded in a biopsychosocial perspective, these findings suggest that malnutrition — a modifiable biological factor — may represent a shared biological correlate where structural and functional vulnerabilities cluster with mental health outcomes. These results underscore the importance of targeting nutritional status as part of integrated, multi-domain interventions aimed at supporting mental health in vulnerable rural populations. Rigorous longitudinal and prospective designs are warranted to examine the directionality and potential causal nature of these associations, and to guide public health strategies that combine individual-level nutritional care with structural improvements in oral health services and food security.

## Data Availability

The original contributions presented in the study are included in the article/supplementary material, further inquiries can be directed to the corresponding author.
